# Toxoplasma gondii Extends the Life Span of Infected Human Neutrophils by Inducing Cytosolic PCNA and Blocking Activation of Apoptotic Caspases

**DOI:** 10.1128/mBio.02031-20

**Published:** 2021-01-26

**Authors:** Tatiane S. Lima, Sharmila Mallya, Allen Jankeel, Ilhem Messaoudi, Melissa B. Lodoen

**Affiliations:** a Department of Molecular Biology and Biochemistry, University of California, Irvine, Irvine, California, USA; b Institute for Immunology, University of California, Irvine, Irvine, California, USA; University of Pittsburgh

**Keywords:** *Toxoplasma gondii*, neutrophil, apoptosis, immune evasion, caspase, host-parasite interaction, immunity, parasite, pathogenesis

## Abstract

Toxoplasma gondii is an intracellular protozoan parasite that has the remarkable ability to infect and replicate in neutrophils, immune cells with an arsenal of antimicrobial effector mechanisms. We report that T. gondii infection extends the life span of primary human peripheral blood neutrophils by delaying spontaneous apoptosis, serum starvation-induced apoptosis, and tumor necrosis alpha (TNF-α)-mediated apoptosis. T. gondii blockade of apoptosis was associated with an inhibition of processing and activation of the apoptotic caspases caspase-8 and -3, decreased phosphatidylserine exposure on the plasma membrane, and reduced cell death. We performed a global transcriptome analysis of T. gondii-infected peripheral blood neutrophils using RNA sequencing (RNA-Seq) and identified gene expression changes associated with DNA replication and DNA repair pathways, which in mature neutrophils are indicative of changes in regulators of cell survival. Consistent with the RNA-Seq data, T. gondii infection upregulated transcript and protein expression of PCNA, which is found in the cytosol of human neutrophils, where it functions as a key inhibitor of apoptotic pro-caspases. Infection of neutrophils resulted in increased interaction of PCNA with pro-caspase-3. Inhibition of this interaction with an AlkB homologue 2 PCNA-interacting motif (APIM) peptide reversed the infection-induced delay in cell death. Taken together, these findings indicate a novel strategy by which T. gondii manipulates cell life span in primary human neutrophils, which may allow the parasite to maintain an intracellular replicative niche and avoid immune clearance.

## Abstract (precis)

Toxoplasma gondii is an obligate intracellular parasite that can cause life-threatening disease in immunocompromised individuals and in the developing fetus. Interestingly, T. gondii has evolved strategies to successfully manipulate the host immune system to establish a productive infection and evade host defense mechanisms.

## INTRODUCTION

Toxoplasma gondii is an obligate intracellular parasite from the Apicomplexa phylum. It infects an estimated 25 to 30% of the global human population, and although most infections are mild or asymptomatic, T. gondii can cause life-threatening disease in immunocompromised individuals and the developing fetus (1). Protective immunity against T. gondii is largely mediated by a strong T cell response; however, the innate immune system is also critical for host defense against this parasite. Accordingly, monocytes, neutrophils, dendritic cells, and natural killer cells have been recognized as important players in the control of T. gondii infection (2–4).

As a foodborne pathogen, T. gondii enters the body and establishes initial infection in the small intestine, from which the parasite disseminates throughout the body. Neutrophils are rapidly recruited to sites of acute T. gondii infection in mice and are known to be infected and serve as a niche of replication for T. gondii (5–7). Neutrophils produce interleukin-12 (IL-12), tumor necrosis factor alpha (TNF-α), and interferon gamma (IFN-γ) (8–10) during T. gondii infection. They also release neutrophil extracellular traps (NETs) in response to the parasite (11) and contribute to dendritic cell activation (12). However, the parasite also appears to employ countermeasures to evade host immunity, as T. gondii infection of neutrophils inhibits lipopolysaccharide (LPS)-induced interleukin 1β (IL-1β) production (13), potentially as a means of immune evasion and dampening of host defense mechanisms.

Host cell manipulation and immune evasion are common strategies observed in apicomplexan parasites. Indeed, T. gondii is well known for having evolved mechanisms to successfully manipulate the host immune system to establish a productive infection and maintain an optimal replicative niche (14, 15). Apoptosis is a form of programmed cell death that can contain the proliferation of intracellular pathogens by depriving them of their intracellular niche. However, in T. gondii-infected cells, both the cell-intrinsic (mitochondrial) and -extrinsic (death receptor-mediated) pathways of apoptosis are disrupted (16, 17). Although the first observation that T. gondii inhibits host cell apoptosis was described over 20 years ago (18), the mechanisms involved in this effect have not been fully elucidated and may vary in different types of host cells. Notably, however, inhibition of the apoptotic caspases, including caspases-8, -9, -3, and -7, by T. gondii was found to be a common step in the parasite’s ability to block apoptosis in different cell lines and mouse primary cells (19–24).

Neutrophils are short-lived cells with an estimated blood half-life of 13 to 19 h in humans (25). As neutrophils age, they exit the blood and enter the liver, spleen, and bone marrow, where they undergo spontaneous apoptosis, thus maintaining homeostatic levels of neutrophils in the blood (26–28). In addition to exhibiting spontaneous apoptosis, neutrophils can engage the intrinsic or extrinsic pathways of apoptosis. Although neutrophils contain low levels of cytochrome *c*, due to reduced numbers of mitochondria, they do require cytochrome *c* to activate apoptotic caspases during the intrinsic pathway (29, 30). The extrinsic pathway of apoptosis is activated as a result of death receptor signaling during neutrophil exposure to members of the tumor necrosis factor (TNF) family, such as Fas ligand (FASL) or TNF-α (31, 32).

Here, we demonstrate that T. gondii extends the life span of human peripheral blood neutrophils during infection by inducing the expression of proliferating cell nuclear antigen (PCNA), a key inhibitor of apoptotic caspases. T. gondii infection also increased PCNA interaction with pro-caspase-3 in the cytosol, preventing caspase-3 activation and inhibiting neutrophil apoptosis. This strategy may allow the parasite to preserve its intracellular niche for replication, thereby enhancing its survival in infected cells.

## RESULTS

### T. gondii infection delays the onset of spontaneous apoptosis in primary human neutrophils.

**FIG 1:**
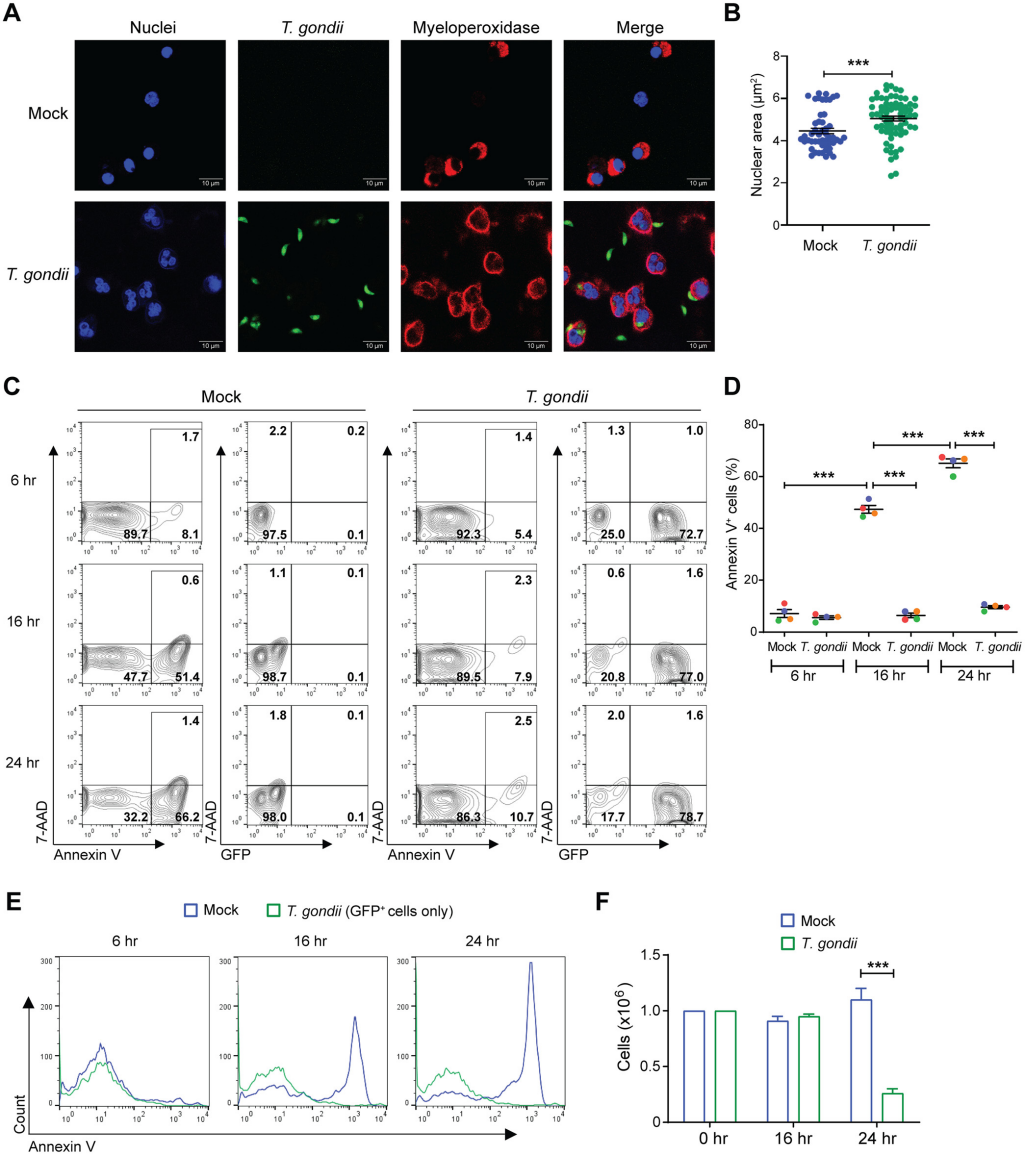
Delay in spontaneous apoptosis of T. gondii-infected human neutrophils. Neutrophils were mock infected or infected with GFP-expressing T. gondii. (A) At 16 h, cells were stained for myeloperoxidase (MPO) and nuclei (DAPI) and analyzed by confocal microscopy (magnification, ×63). Scale bars, 10 μM. (B) The nuclear area was quantified using FIJI. (C) Cells were stained with annexin V and 7-AAD for flow cytometry at 6, 16, and 24 h to identify apoptotic cells (annexin V^+^), dead cells (annexin^+^, 7-AAD^+^), and infected cells (GFP^+^). (D) Percentage of annexin V^+^ cells in each condition. (E) Annexin V levels in GFP^+^ (infected) cells at 6, 16, and 24 h. (F) Viable (trypan blue-negative) cell counts at 0, 16, and 24 h. Values are expressed as means, and error bars reflect standard error of the mean (SEM). ***, *P < *0.001 (unpaired *t* test in panel B and one-way ANOVA, followed by a Tukey posttest in panels D and F). (A) Representative images from 3 independent experiments. (B) We analyzed a total of 75 cells per condition from 3 independent experiments. (C and E) Representative flow cytometry results from 4 independent experiments. (D and F) Experiments were performed four times with independent blood donors, and the combined data are shown.

To evaluate the effect of T. gondii infection on human neutrophil apoptosis, neutrophils were isolated from the peripheral blood of healthy donors through density gradient centrifugation. This protocol resulted in greater than 90% neutrophils, as previously reported (13). In culture, aging neutrophils exhibit classical signs of apoptosis, such as cell shrinkage and condensation of nuclear heterochromatin (33–35). Consistent with these previous reports, neutrophils that were “mock” infected with media supplemented with fetal bovine serum (FBS) displayed cell and nuclear shrinkage after 16 h of culture, as observed by immunofluorescence staining of the cell nucleus and the neutrophil-specific myeloperoxidase protein (Fig. 1A, top row). In contrast, neutrophils infected with type I (RH strain) T. gondii and cultured under the same conditions maintained the morphology of healthy live cells (Fig. 1A, bottom row). The quantification of nuclear areas for mock- and T. gondii*-*infected neutrophils reflected this difference (Fig. 1B).

In addition to changes in neutrophil morphology, a hallmark of the onset of apoptosis is the exposure of phosphatidylserine (PS) on the outer membrane of the cell, which can be detected by staining with annexin V (36). In addition, 7-aminoactinomycin D (7-AAD) can be used to detect late apoptotic and dead cells (37). By examining annexin V staining of mock-infected neutrophils after 6, 16, or 24 h of culture, we found that PS exposure increased over time, and greater than 50% of the cells were positive for annexin V after 16 h (Fig. 1C and D). In parallel, neutrophils were infected with green fluorescent protein (GFP)-expressing T. gondii, which allowed us to determine the infection efficiency over time and the levels of annexin V specifically in the infected (GFP^+^) or uninfected bystander populations. Our experimental conditions resulted in greater than 70% infection efficiency and showed that the vast majority of GFP^+^ infected cells were negative for annexin V and 7-AAD (Fig. 1C and E). Surprisingly, culturing neutrophils for 24 h with 10% fetal bovine serum (FBS) resulted in very few 7-AAD^+^ dead cells (Fig. 1C). To evaluate the numbers of cells detectable in the cultures, total cell counts with trypan blue were performed immediately after the cells were plated and after 16 and 24 h in culture. Interestingly, there was no change in the total numbers of mock-infected cells over time, indicating that although the cells exhibited molecular signs of the onset of apoptosis (annexin V detection of PS exposure), they persisted in the culture. The loss of T. gondii*-*infected cells at 24 h was due to infected neutrophils lysing as a result of overwhelming parasite burden (Fig. 1F), consistent with prior reports by our lab and others that neutrophils are a replicative niche for T. gondii (13, 38, 39). Collectively, these data demonstrate that T. gondii infection delays the onset of the spontaneous apoptosis of primary human neutrophils.

### T. gondii infection inhibits serum starvation-induced and TNF-α-induced cell death in primary human neutrophils.

**FIG 2:**
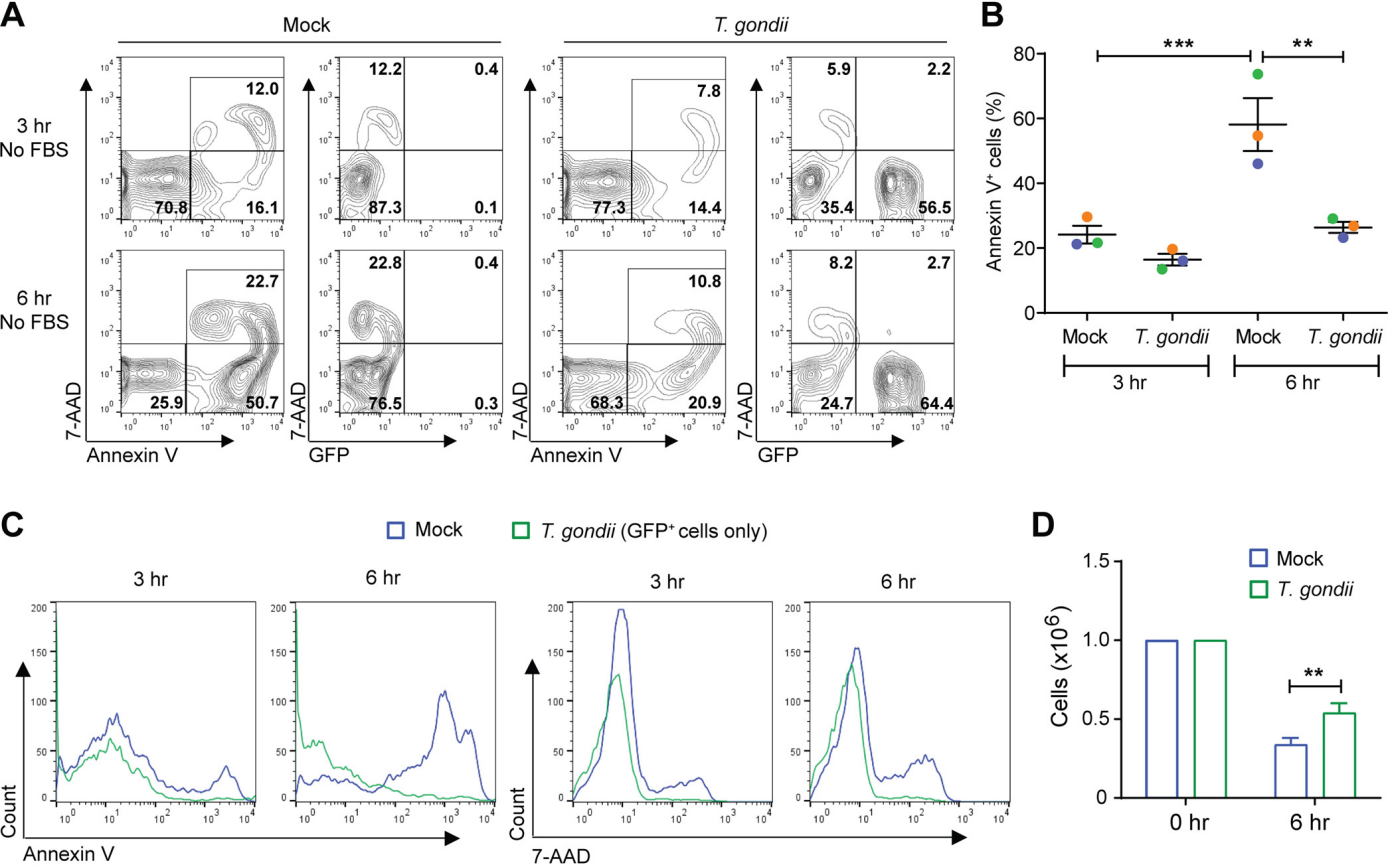
Starvation-induced cell death is inhibited in T. gondii-infected human neutrophils. Neutrophils were mock infected or infected with GFP-expressing T. gondii in medium without FBS. (A) Cells were stained with annexin V and 7-AAD for flow cytometry at 3 and 6 h to identify apoptotic cells (annexin V^+^), dead cells (annexin^+^, 7-AAD^+^), and infected cells (GFP^+^). (B) Percentage of annexin V^+^ cells in each condition. (C) Annexin V and 7-AAD fluorescence in GFP^+^ (infected) cells at 3 and 6 h. (D) Viable (trypan blue-negative) cell counts at 0 and 6 h. Values are expressed as means, and error bars reflect the SEM. **, *P < *0.01; ***, *P < *0.001 (one-way ANOVA, followed by a Tukey posttest). (A and C) Representative flow cytometry results from 3 independent experiments are shown. (B and D) Experiments were performed three times with independent blood donors, and the combined data are shown.

We next evaluated the impact of T. gondii infection on serum starvation-induced apoptosis, which leads to rapid neutrophil death. In this model, primary human neutrophils were cultured for 3 or 6 h without FBS. Within 6 h of culture without serum, on average, approximately 60% of mock-infected neutrophils were in the early (annexin V^+^) or late (annexin V^+^ 7-AAD^+^) stage of apoptosis (Fig. 2A and B). In contrast, less than 35% of T. gondii-infected neutrophils had progressed to these stages of apoptosis (Fig. 2A and B). As with the model of spontaneous apoptosis, nearly all T. gondii-infected GFP^+^ cells were negative for annexin V and 7-AAD (Fig. 2C). Total cell counts with trypan blue confirmed that T. gondii infection contributed to cell survival, as more T. gondii-infected cells persisted in the cultures than in mock-infected neutrophils during serum starvation (Fig. 2D).

**FIG 3:**
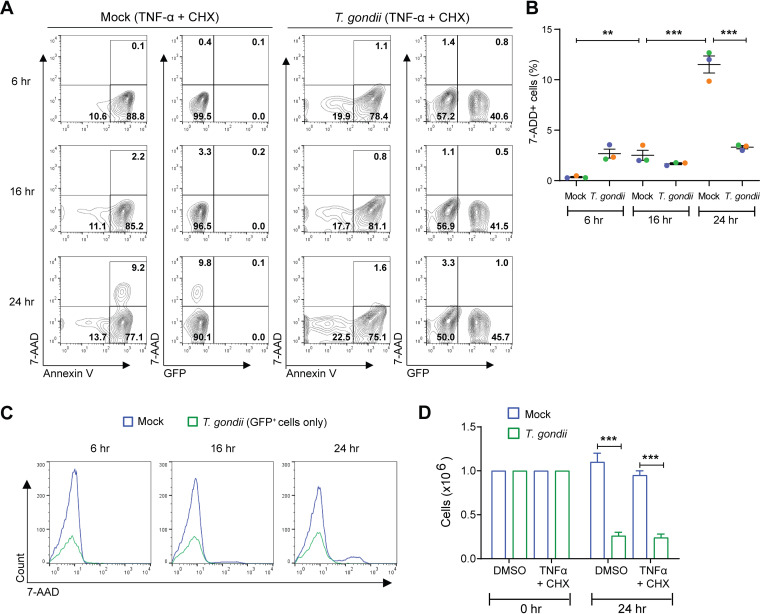
TNF-α-induced apoptosis is inhibited in T. gondii-infected human neutrophils. Neutrophils were either mock infected or infected with GFP-expressing T. gondii for 1 h and then treated with DMSO (see the data presented in Fig. 1C) or TNF-α (20 ng/ml) plus CHX (2.5 ng/ml). Cells were cultured for a total of 6, 16, or 24 h. (A) Cells were stained with annexin V and 7-AAD for flow cytometry to identify apoptotic cells (annexin V^+^), dead cells (annexin^+^, 7-AAD^+^), and infected cells (GFP^+^). (B) Percentages of 7-AAD^+^ cells were determined. (C) 7-AAD fluorescence in GFP^+^ (infected) cells at 6, 16, and 24 h. (D) Viable (trypan blue-negative) cell counts at 0 and 24 h. Values are expressed as means, and error bars reflect the SEM. **, *P < *0.01; ***, *P < *0.001 (one-way ANOVA, followed by a Tukey posttest). (A and C) Representative flow cytometry from 3 independent experiments are shown. (B and D) Experiments were performed three times with independent blood donors, and the combined data are shown.

The ligation of death receptors, such as TNF receptor-1 (TNFR1), results in the extrinsic pathway of apoptosis (40, 41). To test the effect of T. gondii infection on this pathway, human neutrophils were first incubated with T. gondii for 1 h to allow infection, followed by treatment with TNF-α to induce apoptosis. Cycloheximide (CHX), an inhibitor of protein biosynthesis, was added with TNF-α to prevent the upregulation of antiapoptotic proteins that occurs in cells treated with TNF-α (42, 43). The combination of TNF-α and CHX rapidly initiated apoptosis, as greater than 80% of the cells were annexin V positive within 6 h (Fig. 3A). Interestingly, in this model, T. gondii infection did not prevent the onset of apoptosis (annexin V^+^ 7-AAD^–^ cells); however, parasite infection significantly delayed the progression to late apoptosis: on average, 3.3% of cells in the infected culture were 7-AAD^+^ compared to 11.5% in the mock-infected culture at 24 h postinfection (hpi) (Fig. 3A and B). Again, nearly all GFP^+^, T. gondii-infected cells were negative for 7-AAD (Fig. 3C), and total cell counts at 24 h reflected a loss of infected neutrophils due to parasite lysis of the cells (Fig. 3D). Taken together, these findings indicate that T. gondii prolongs the life span of human neutrophils by delaying the apoptosis of these cells in three models of apoptosis.

### T. gondii infection inhibits caspase-8 and caspase-3 cleavage and activity in primary human neutrophils.

**FIG 4:**
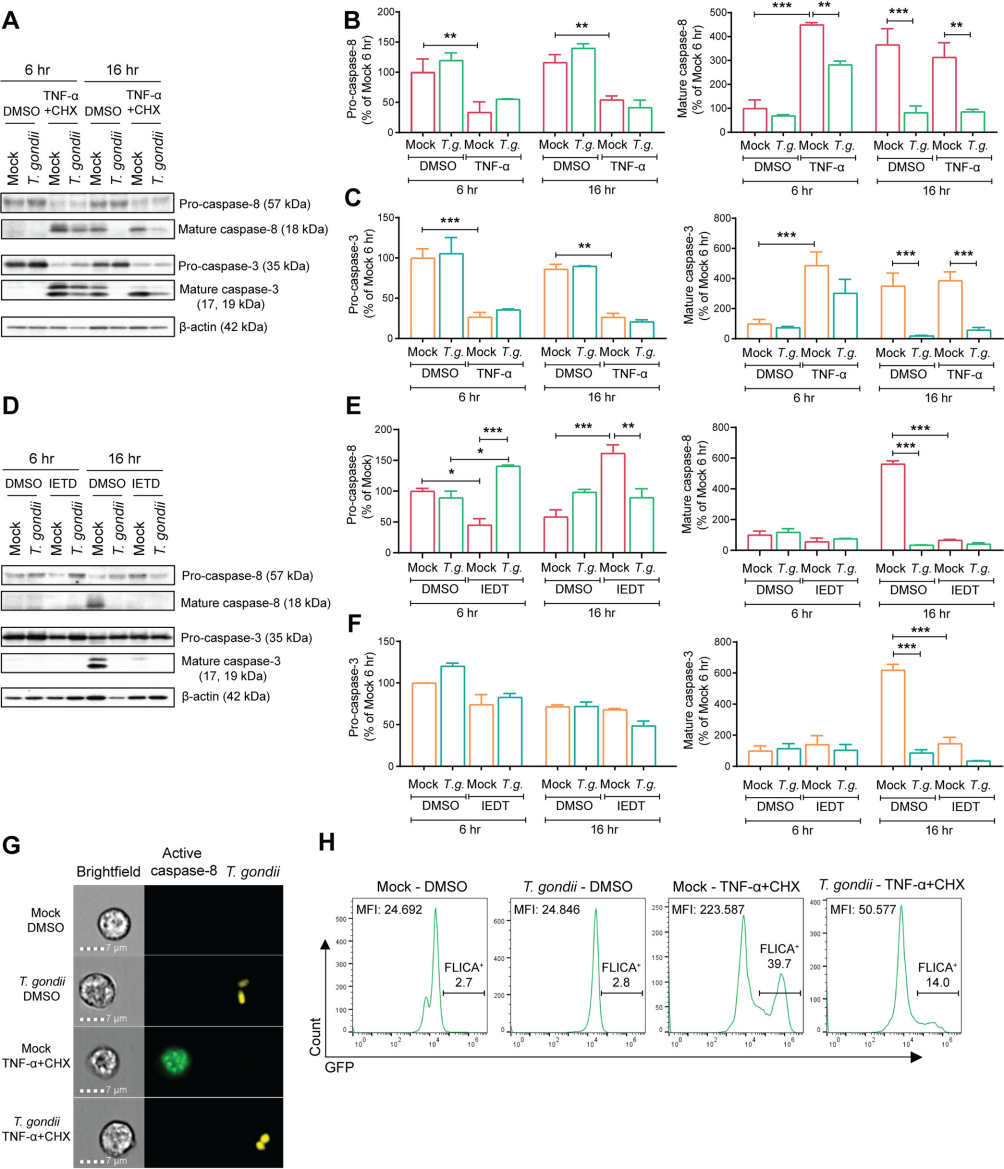
The processing and activity of caspase-8 and caspase-3 are inhibited in T. gondii-infected human neutrophils. (A to C) Neutrophils were either mock infected or infected with GFP-expressing T. gondii for 1 h, treated with DMSO or TNF-α plus CHX, and cultured for a total of 6 or 16 h. Pro- and cleaved caspase-8, pro- and cleaved caspase-3, and β-actin in the cell lysate were visualized by Western blotting (A) and quantified by densitometry (B and C). (D to F) Neutrophils were treated with the caspase-8 inhibitor Z-IEDT-FMK (100 μM) or DMSO and either mock infected or infected with T. gondii for 6 or 16 h. Pro- and cleaved caspase-8, pro- and cleaved caspase-3, and β-actin in the cell lysate were visualized by Western blotting (D) and quantified (E and F). (G) Neutrophils were mock infected or infected with tdTomato-expressing T. gondii for 16 h and treated with TNF-α and CHX as described for panel A. Caspase-8 activity was detected by the FAM-FLICA probe and analyzed by ImageStream flow cytometry. (H) Percentage of FLICA^+^ cells and mean fluorescence intensity (MFI) of GFP (FLICA probe) in each experimental group. Values are expressed as means, and error bars reflect the SEM. *, *P < *0.05; **, *P < *0.01; ***, *P < *0.001 (one-way ANOVA, followed by a Tukey posttest). Representative images from 4 (A) and 3 (D and G) independent experiments are shown. Data reflect combined results of four (B and C) and three (E to H) experiments with different blood donors.

Caspases are proteases well known for their role in controlling cell death and inflammation. The apoptotic caspases are divided into the initiator caspases, such as caspase-8, and effector caspases, such as caspase-3, depending on specific protein interaction domains present in the N terminus that mediate their activation (44, 45). Due to their importance in regulating apoptosis, we investigated the cleavage and activity of caspase-8 and caspase-3 during spontaneous and TNF-α-induced apoptosis of T. gondii-infected neutrophils. We examined pro- and cleaved caspase-8 and caspase-3 by Western blotting in cells that were treated with TNF-α plus CHX or the vehicle control dimethyl sulfoxide (DMSO). In DMSO-treated cells, cleavage of pro-caspase-8 and pro-caspase-3 into mature caspases occurred spontaneously after 16 h in culture and was strongly inhibited in T. gondii-infected neutrophils (Fig. 4A, B, and C). The processing of caspase-8 and caspase-3 induced by TNF-α plus CHX occurred more rapidly and could be detected after 6 h of treatment. T. gondii infection also decreased the processing of these two apoptotic caspases in TNF-α- and CHX-treated cells, and this effect was more pronounced at the later time point of 16 h (Fig. 4A, B, and C).

To confirm a role for the initiator caspase-8 on effector caspase-3 processing during the spontaneous apoptosis of neutrophils, we used Z-IETD-FMK (Z-Ile-Glu-Thr-Asp-fluoromethylketone), a cell-permeable inhibitor that binds to the active site of caspase-8 and prevents substrate interaction (46). As expected, cleavage of caspase-8 was inhibited in IETD-treated neutrophils compared to cleavage in the DMSO control (Fig. 4D and E). In addition, caspase-3 cleavage was also inhibited in IETD-treated cells (Fig. 4D and F), indicating that its cleavage occurs downstream of caspase-8 activity during spontaneous apoptosis of human neutrophils.

To examine caspase-8 activity, we used the FAM-LETD-FMK FLICA (Carboxyfluorescein-LETD-FMK Fluorochrome-Labeled Inhibitor of Caspase) probe, which is cell permeable and binds specifically to active caspase-8 (47). Neutrophils were analyzed by ImageStream flow cytometry (Fig. 4G), and caspase-8 activity was detected in less than 3% of uninfected (mock) neutrophils or in those infected with tdTomato-expressing T. gondii (Fig. 4H). The treatment of neutrophils with TNF-α plus CHX induced high levels of active caspase-8, as shown by the numerous and intense FLICA specks within the cells (Fig. 4G), and nearly 40% of the cells became FLICA^+^ (Fig. 4H). T. gondii infection reduced TNF-α-induced caspase-8 activity, as indicated in the representative images (Fig. 4G), and by the reduced mean fluorescence intensity (MFI) of GFP and percentage of FLICA^+^ cells (Fig. 4H). These findings indicate that T. gondii infection inhibits the processing and activity of apoptotic caspases in neutrophils, which may contribute to the decrease in PS exposure and cell death observed during T. gondii infection of these cells.

### Neutrophil transcriptional response to T. gondii infection.

**FIG 5:**
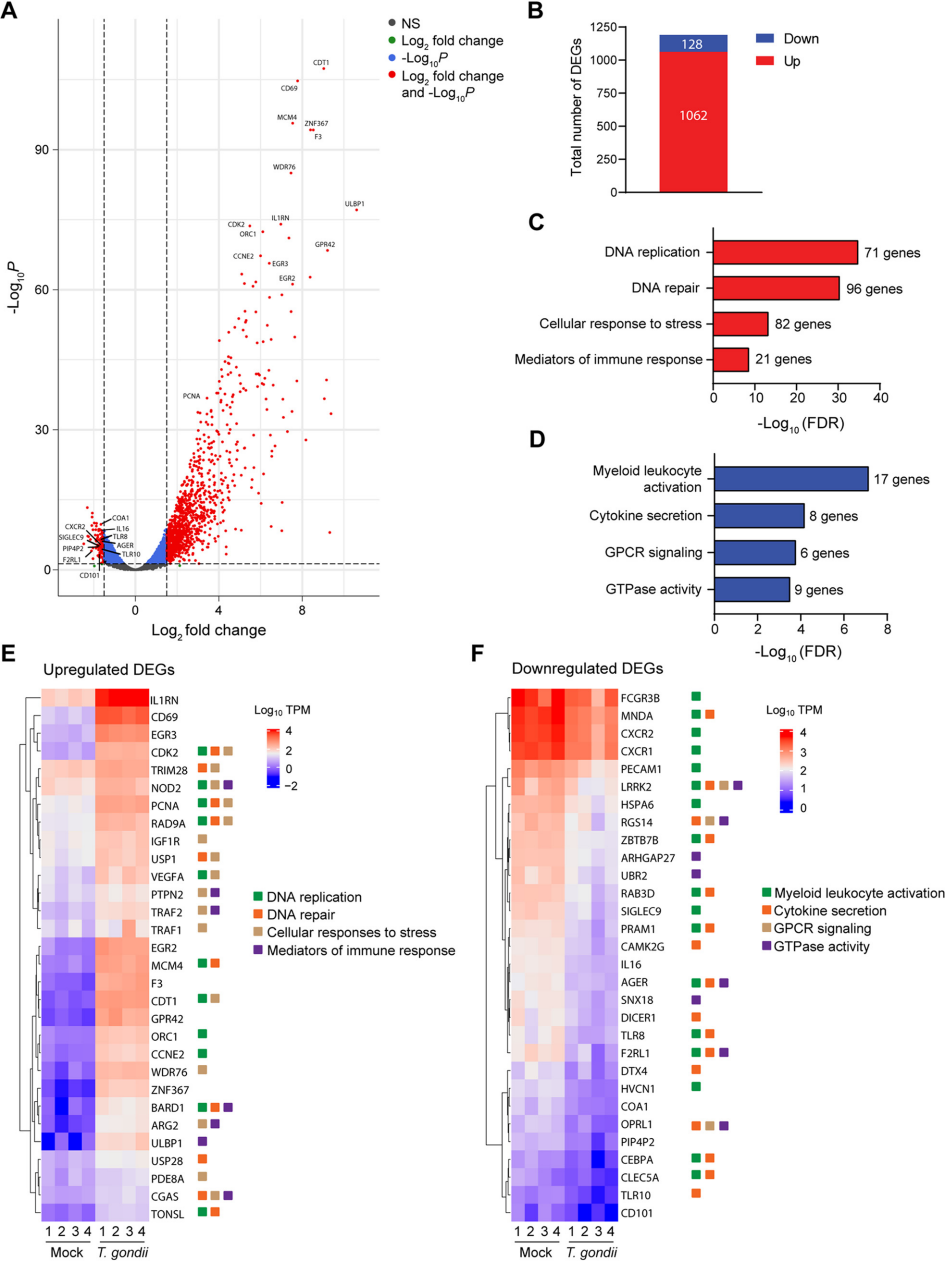
Neutrophil transcriptional response to T. gondii infection. Neutrophils were mock infected or infected with T. gondii for 4 h, and RNA-Seq was performed on samples from 4 independent donors. (A) Volcano plot depicting total gene expression changes in neutrophils, with red representing significantly differentially expressed genes (DEGs) (fold change > 1.5, false discovery rate (FDR) < 0.05, and reads per kilobase million [RPKM] > 1) and black representing nonsignificantly (NS) changed genes. The top 15 upregulated and 10 downregulated DEGs are labeled. (B) Total number of DEGs up- or downregulated by T. gondii infection. (C and D) Top four gene ontology (GO) terms for gene sets with increased (C) or decreased (D) transcript abundance. The number of genes within each GO term is shown. (E and F) Heatmaps show the expression of 30 upregulated (E) and downregulated (F) DEGs. Normalized numbers of transcripts per million (TPM) are shown. The range of colors is based on scaled and centered TPM values of the entire set of genes (red represents high expression, and blue represents low expression). The genes mapped to the top four regulated pathways are indicated by colored squares.

To further investigate the mechanisms associated with the delay in apoptosis of human neutrophils, we examined the transcriptional response of these cells to T. gondii infection by RNA sequencing (RNA-Seq). We found 1,190 protein-coding genes that were differentially expressed between mock-infected and T. gondii-infected neutrophils at 4 hpi (fold change >1.5; false discovery rate [FDR] <0.05; and reads per kilobase million [RPKM] >1) (Fig. 5A). Most of the differentially expressed genes (DEGs) were upregulated by T. gondii infection: there were 1,062 genes with increased transcripts and only 128 with decreased transcripts in response to infection (Fig. 5B, and see Fig. S1 in the supplemental material). Among the most highly expressed genes induced by T. gondii infection were *CDT1* (chromatin licensing and DNA replication factor 1), *CD69*, *MCM4* (DNA replication licensing factor MCM4), *ZNF367* (zinc finger protein 367), and *F3* (tissue factor) (Fig. 5A).

10.1128/mBio.02031-20.1 FIG S1 Global DEGs in T. gondii-infected neutrophils. Neutrophils were mock infected or infected with T. gondii for 4 h, and RNA-Seq was performed on samples from 4 independent donors. Heatmaps represent the expression of all upregulated and downregulated DEGs. Normalized numbers of TPM (transcripts per million) are shown. The range of colors is based on scaled and centered TPM values of the entire set of genes (red represents high expression, and blue represents low expression). Download FIG S1, TIF file, 1.8 MB. Copyright © 2021 Lima et al. 2021 Lima et al. This content is distributed under the terms of the Creative Commons Attribution 4.0 International license.

Gene ontology analysis was conducted to identify biological processes associated with gene sets that were up or downregulated during infection. Based on this analysis, upregulated gene sets were enriched in DNA replication and DNA repair processes, with a total of 167 DEGs with increased transcript abundance in infected cells mapping to these pathways (Fig. 5C). Since neutrophils are terminally differentiated cells, it is notable that several of the DEGs within these processes, including *PCNA* (proliferating cell nuclear antigen), *CDK2* (cyclin-dependent kinase 2), and *BARD1* (BRCA1-associated RING domain protein 1), not only regulate DNA replication and DNA repair but also are involved in coordinating apoptosis and cell survival (48–50) (Fig. 5E). In addition, many genes associated with the cellular response to stress and the immune response were upregulated in T. gondii-infected neutrophils (Fig. 5C and E). The functional enrichment of DEGs with decreased transcript abundance revealed that T. gondii infection mainly downregulated genes associated with leukocyte activation, cytokine secretion, G protein-coupled receptor (GPCR) signaling, and GTPase activity (Fig. 5D and F). Among these downregulated genes were immune receptors and chemokines, including *AGER* (receptor for advanced glycation end products), *SIGLEC9* (sialic acid-binding immunoglobulin-like lectin 9), and *CXCR2* (interleukin 8 receptor β).

### An increase in cytosolic PCNA prevents activation of caspase-3 in T. gondii-infected neutrophils.

**FIG 6:**
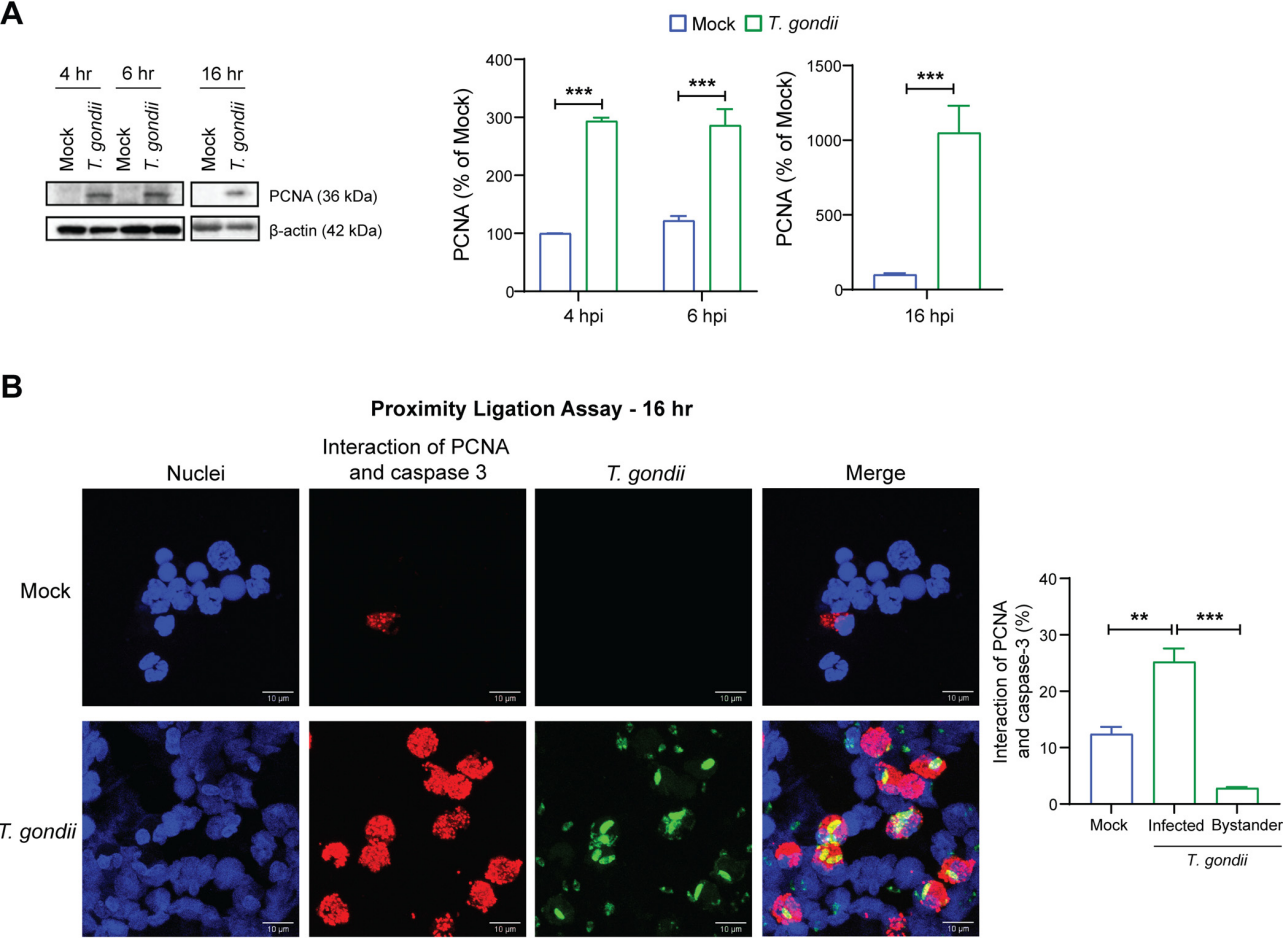
Effect of T. gondii infection on PCNA expression and its interaction with pro-caspase-3 in human neutrophils. (A) Neutrophils were mock infected or infected with GFP-expressing T. gondii. PCNA and β-actin in the cell lysate were detected and quantified by Western blotting at 4, 6, and 16 h. (B) Neutrophils were infected on coverslips with T. gondii for 16 h. A proximity ligation assay was used to detect the interaction of PCNA with caspase-3, and cells were counterstained with Hoechst dye for confocal microscopy (magnification, ×63). Scale bars, 10 μM. Bar graphs represent the percentages of cells with PCNA-caspase-3 interactions under each condition, including directly infected (GFP^+^) cells and bystander cells. Values are expressed as means, and error bars reflect the SEM. **, *P < *0.01; ***, *P < *0.001 (one-way ANOVA, followed by a Tukey posttest). (A and B) Representative blots and images from 3 independent experiments are shown. Experiments were performed three times with independent blood donors, and the combined data are shown.

PCNA is a key regulator of neutrophil survival. In neutrophils, PCNA is localized exclusively in the cytoplasm and constitutively associates with pro-caspases, preventing their activation (48, 51). Since T. gondii infection upregulated PCNA gene expression (Fig. 5E), we hypothesized that PCNA may contribute to the mechanism by which T. gondii inhibits apoptosis in human neutrophils. To investigate this possibility, we examined PCNA protein levels in T. gondii-infected neutrophils. T. gondii infection increased PCNA protein levels as early as 4 hpi in a comparison with mock-infected neutrophils, and this increase persisted to 16 hpi (Fig. 6A). We next examined the degree to which PCNA interacted with pro-caspase-3 in mock- or T. gondii-infected neutrophils. For these studies, we used the proximity ligation assay (PLA), which enables the detection of proteins of interest that are within 40 nm and is, therefore, able to detect protein-protein interactions (52). We observed that T. gondii infection significantly increased the interaction between PCNA and pro-caspase-3 in human neutrophils. Interestingly, in the infected cell culture, nearly all the cells in which PCNA interacted with pro-caspase-3 were cells with intracellular T. gondii, and this interaction was detected in very few uninfected bystander cells (Fig. 6B). These data indicate that T. gondii infection induces PCNA mRNA and protein expression and leads to increased binding between PCNA and pro-caspase-3, potentially inhibiting the processing and activation of caspase-3 in T. gondii-infected neutrophils.

### Inhibition of the APIM motif from PCNA induces caspase processing and cell death in T. gondii-infected neutrophils.

**FIG 7:**
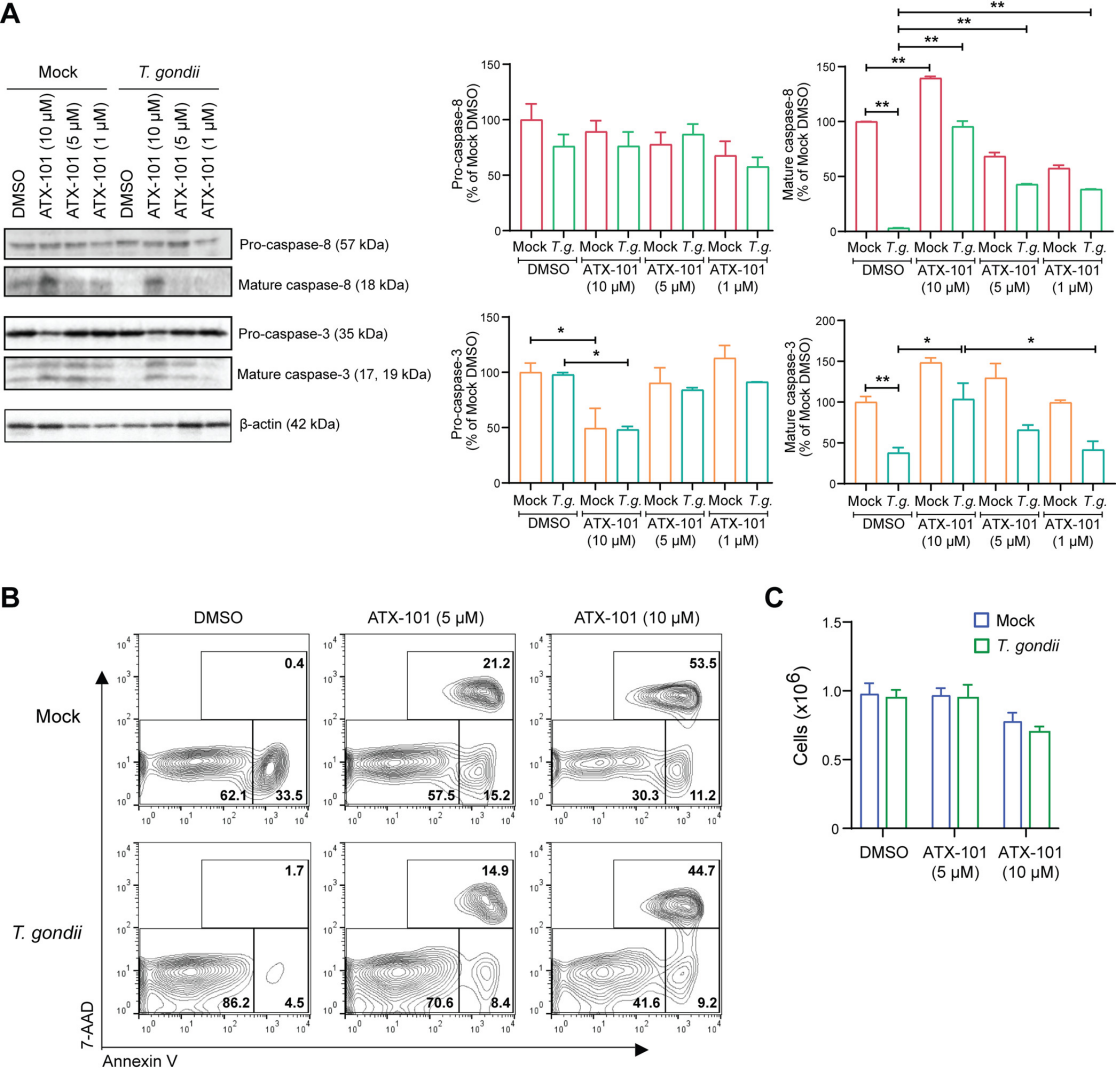
Effect of the PCNA inhibitor ATX-101 in caspase processing and cell death in T. gondii-infected human neutrophils. Neutrophils were mock infected or infected with GFP-expressing T. gondii in the presence of the APIM-containing peptide ATX-101 (1, 5, or 10 μM) or the vehicle control for 8 h. (A) Pro- and cleaved caspase-8, pro- and cleaved caspase-3, and β-actin in the cell lysate were visualized and quantified by Western blotting. (B) Cells were stained with annexin V and 7-AAD for flow cytometry to identify apoptotic cells (annexin V^+^) and dead cells (annexin^+^, 7-AAD^+^). (C) Viable (trypan blue-negative) cell counts at 8 h. Values are expressed as means, and error bars reflect the SEM. *, *P < *0.05; **, *P < *0.01 (one-way ANOVA, followed by a Tukey posttest). (A and B) Representative Western blots and flow from 3 independent experiments are shown. (A and C) Experiments were performed three times with independent blood donors, and the combined data are shown.

Several proteins interact with PCNA through the AlkB homologue 2 PCNA-interacting motif (APIM), including apoptosis-regulating proteins (53). Since T. gondii infection increased the association between PCNA and pro-caspase-3, we next investigated if we could inhibit this interaction, thereby increasing the processing of caspase-3, by using an APIM motif-containing peptide. We targeted PCNA with ATX-101, which is a cell-permeable, APIM-containing peptide that inhibits the interaction of PCNA with other proteins through this motif (54, 55). After treatment of neutrophils with the DMSO vehicle control or with increasing concentrations of ATX-101, we analyzed the processing of caspase-8 and caspase-3 in mock- and T. gondii-infected neutrophils at 8 hpi. The treatment of cells with the APIM-containing peptide induced cleavage of both caspases in mock- and T. gondii-infected neutrophils, indicating reduced inhibition of caspase cleavage and activity in the presence of the peptide (Fig. 7A). The effect of the inhibitor on mock-infected cells suggests that PCNA also contributes to maintaining the viability of uninfected neutrophils. Consistent with these findings on caspase cleavage, ATX-101 treatment led to increased PS exposure and eventual cell death in both mock- and T. gondii-infected neutrophils, as detected by staining with annexin V and 7-AAD and analysis by flow cytometry (Fig. 7B). To exclude the possibility of a loss of dead cells during fluorescence-activated cell sorting (FACS) staining, total cell counts with trypan blue were measured in the presence and absence of ATX-101 (Fig. 7C). Taken together, these findings demonstrate that the interaction between PCNA and pro-caspases through the APIM motif is necessary to prevent caspase processing and cell death during the apoptosis of T. gondii-infected neutrophils.

## DISCUSSION

Each day approximately 100 billion neutrophils are produced in the bone marrow of the average human adult, and neutrophil homeostasis is maintained by an orchestrated balance between neutrophil development, mobilization from the bone marrow, migration into peripheral tissues, cell aging, and death (25). Neutrophils are terminally differentiated cells with a short life span, and spontaneous apoptosis is the predominant cell death pathway in these cells. During spontaneous apoptosis, aged peripheral blood neutrophils migrate into the liver, spleen, and bone marrow, where they are cleared by macrophages (26). Our data show a novel strategy of host cell manipulation in which T. gondii infection extends the life span of primary human neutrophils by delaying apoptosis.

T. gondii is widely known for its ability to successfully manipulate the host immune system to establish a productive infection and maintain an optimal replicative niche (14, 15). Indeed, both type I and type II T. gondii can inhibit the apoptosis of several murine and human cell lines, which may help the parasite to preserve its intracellular niche (16, 56). However, limited information is available on the effect of T. gondii infection on the apoptosis of primary human immune cells.

The process of apoptosis is characterized by morphological changes that include cell shrinkage, cytoplasmic condensation, and condensation of nuclear heterochromatin (33–35). In the final stages of apoptosis, the entire cell is condensed and reorganized into apoptotic bodies, a term introduced in 1972 (57). However, before these morphological changes can be visualized by microscopy, changes in the composition of the plasma membrane indicate the onset of apoptosis. Phospholipids are distributed asymmetrically in the plasma membrane, and PS is restricted to the cytoplasmic leaflet of the plasma membrane. The loss of phospholipid asymmetry and exposure of PS on the cell surface was first observed in apoptotic lymphocytes (58–60); however, it is now considered a ubiquitous feature of apoptotic cells, including neutrophils (61). Interestingly, unlike with uninfected neutrophils, we found that T. gondii-infected neutrophils maintained the morphology of healthy neutrophils for at least 16 h in culture. By utilizing fluorochrome-labeled annexin V, which binds to PS in a calcium-dependent manner (62, 63), we also detected low levels of PS in the plasma membrane of neutrophils infected with GFP-expressing T. gondii, whereas uninfected neutrophils expressed higher levels of PS. Most neutrophils in our cell cultures were infected, and the few events in which PS was exposed in the plasma membrane were in noninfected cells, suggesting that the delay in the onset of apoptosis was dependent on the presence of the parasite inside the cell.

Although apoptosis was ongoing in most mock-infected neutrophils cultured for 24 h, surprisingly, very few dead cells were found during analysis with a cell viability dye. Under these circumstances, we then tested the impact of T. gondii infection in two different *in vitro* models that are characterized by a rapid onset of apoptosis and cell death. When neutrophils were cultured under serum starvation conditions, high levels of apoptotic and dead cells were observed within 3 h. However, T. gondii infection delayed the process of apoptosis and extended the life span of these starved neutrophils. Apoptosis can also be induced through an extrinsic pathway triggered by ligation of death receptors (40, 41). Interestingly, the onset of apoptosis was not prevented or reversed by T. gondii infection in TNF-α-treated neutrophils, but consistent with the two previous models, the entire process was delayed, and cell life span was prolonged. Although the extrinsic pathway of apoptosis was induced by the combination of TNF-α and CHX, an inhibitor of protein biosynthesis that affects both the neutrophil and the parasite, we anticipate that the delay in apoptosis does not require parasite replication, since we observed an inhibition of serum starvation-induced apoptosis within 3 and 6 h after infection, time points that are prior to and just at the onset of parasite replication. Thus, our data suggest that T. gondii affects a common step among the three pathways of apoptosis reported in this study, which results in a delay in the cascade of events involved in apoptotic cell death, potentially extending the parasite’s ability to utilize neutrophils as a niche for replication (5–7, 13).

Caspases are a family of cysteine proteases involved in apoptosis, inflammation, and development. In healthy cells, the apoptotic caspases, such as caspase-8 and caspase-3, localize to the cytosol and are found as inactive pro-enzymes (64, 65). Irrespective of the pathway of apoptosis, caspase cleavage and activation are a common step that triggers a variety of downstream processes to result in many of the biochemical and biophysical changes that occur during apoptosis. Previous studies have demonstrated that both type I and type II strains of T. gondii inhibit the activation of apoptotic caspases in various cell lines (19, 22–24). Consistent with this, we observed inhibition of spontaneous and TNF-α-induced caspase-8 and caspase-3 processing in T. gondii-infected primary human neutrophils. Additionally, the FLICA assay confirmed low levels of enzymatically active caspase-8 in infected cells that were stimulated with TNF-α. Pro-caspase-3 is known as the major physiologic target of caspase-8 (66). Indeed, we confirmed a role for the initiator caspase-8 in caspase-3 processing during spontaneous apoptosis of human neutrophils. Since PS exposure during apoptosis depends on caspase-induced regulation of phospholipid translocases (activation of the Xkr8 scramblase activity and inactivation of the flippase activity of ATP11A and ATP11C) (67), our data suggest that the reduced levels of exposed PS in T. gondii-infected neutrophils is a result of decreased caspase-8 and caspase-3 activity.

To better characterize the mechanisms associated with the delay in apoptosis of human neutrophils, we examined the transcriptional response of these cells to T. gondii infection by RNA-Seq. Functional enrichment of the upregulated genes revealed significant representation of DNA replication and DNA repair processes. Among the list of genes enriched to these processes was *PCNA*, which is also involved in coordinating apoptosis and cell survival (48–50). As neutrophils are terminally differentiated cells, many genes listed in the pathways of DNA replication and repair may, in fact, play a role in neutrophil survival and apoptosis. Similarly, our group and others have also described T. gondii-induced upregulation of genes involved in the cell cycle; however, these findings were reported in dividing cells, such as human foreskin fibroblasts (HFF) (68) and human umbilical vein endothelial cells (HUVEC) (69). Furthermore, T. gondii infection downregulated genes associated with leukocyte activation and cytokine secretion. Interestingly, *SIGLEC9* was highly downregulated in neutrophils upon T. gondii infection. Siglec-9 is expressed predominantly on monocytes and neutrophils (70, 71) and plays an important role in initiating caspase-dependent apoptosis in human neutrophils (72).

PCNA is a critical auxiliary protein in DNA synthesis and repair (73, 74). Interestingly, terminally differentiated neutrophils express PCNA exclusively in their cytosol due to its export from the nucleus to the cytosol during neutrophil differentiation. In mature neutrophils, PCNA interacts with pro-caspases, which prevents their processing and activation, thus regulating neutrophil life spans (48, 51, 75). Accordingly, PCNA levels decline during neutrophil apoptosis and are sustained by prosurvival stimuli, such as granulocyte colony-stimulating factor (G-CSF) (48). In addition to finding the upregulation of *PCNA* transcripts and a sustained increase in PCNA protein during infection of neutrophils, we found that T. gondii significantly increased the interaction between PCNA and pro-caspase-3 in the cytosol of human neutrophils. Thus, our data suggest that T. gondii-induced PCNA expression is involved in preventing caspase processing and activation in infected neutrophils. Moreover, the interaction between PCNA and pro-caspase-3, through the APIM motif, was necessary to prevent caspase processing and apoptosis of T. gondii-infected human neutrophils. To our knowledge, the physiological role of PCNA in cell survival has been described only for human neutrophils; however, a similar role may be present in other terminally differentiated cells with little to no DNA replication or repair. Thus, the effect of T. gondii infection on PCNA expression may contribute to blocking apoptosis in other terminally differentiated cells. Although we were able to characterize PCNA as a central regulator of neutrophil survival during T. gondii infection, the effector protein that triggers this response remain unknown and will be further investigated in follow-up studies. Here, we report the effect of type I (RH) T. gondii on the inhibition of human neutrophil apoptosis; however, previous studies have also indicated that type II T. gondii strains (NTE and Pru) are able to prevent caspase processing and to inhibit the apoptosis of human cell lines (19–21, 24, 76–79). These findings suggest that type II T. gondii may also affect human neutrophil apoptosis through PCNA. Additionally, our group has demonstrated that type I (RH) and II (PA7) T. gondii inhibit IL-1β release from human neutrophils (13), indicating the ability of both strains to manipulate and evade the innate immune response of human host neutrophils.

Collectively, the current findings provide evidence of a novel strategy by which T. gondii manipulates cell life span in peripheral human neutrophils by inducing cytosolic PCNA and inhibiting caspase-8 and caspase-3 processing, which may allow the parasite to preserve its intracellular niche, while replicating and avoiding immune clearance.

## MATERIALS AND METHODS

### Isolation of primary human neutrophils.

Human peripheral blood from healthy donors was provided by the Institute for Clinical and Translational Science (ICTS) at the University of California, Irvine, in accordance with the guidelines and approval of the Institutional Review Board. Neutrophils were isolated as previously described (13). Briefly, freshly collected blood was mixed with 3% dextran (Sigma-Aldrich) in phosphate-buffered saline (PBS) for 20 min (min) at room temperature (RT). The top layer containing leukocytes was transferred to a fresh tube, and the cells were underlaid with 15 ml of Ficoll Paque Plus (GE Healthcare) and centrifuged at 300 × *g* for 20 min at RT. The underlying pellet containing neutrophils and red blood cells (RBC) was suspended in 1× RBC lysis buffer (eBioscience) and incubated for 10 min at RT. Neutrophils were washed in PBS and suspended in RPMI 1640 (HyClone) supplemented with 2 mM l-glutamine (Corning), 100 U ml^−1^ penicillin, and 100 μg ml^−1^ streptomycin (HyClone). As an additional supplement, 10% heat-inactivated fetal bovine serum (FBS) (Omega Scientific) was used in some experiments. Isolated human neutrophils were immediately used for experimentation.

### Parasite culture and neutrophil infections.

Type I T. gondii (RH) tachyzoites constitutively expressing green fluorescent protein (GFP) or tdTomato were maintained in human foreskin fibroblasts (HFF) as previously described (80). Syringe-lysed parasites were passed through a 5.0-μm filter unit to remove host cell debris, and neutrophils were infected at a multiplicity of infection (MOI) of 2. Mock-infected neutrophils were those to which only fresh medium was added. Parasite cell cultures were routinely tested for *Mycoplasma* contamination and confirmed to be negative.

### Stimulators and inhibitors.

TNF-α (R&D Systems) was used at 20 ng ml^−1^ to stimulate the extrinsic apoptosis pathway though activation of TNFR1 (40). The protein synthesis inhibitor CHX was used at 2.5 μg ml^−1^ to prevent the synthesis of antiapoptotic proteins induced by the TNF-α treatment (42). Neutrophils were treated with TNF-α in combination with CHX, or with an equivalent volume of DMSO as the vehicle control, for 6, 16, or 24 h. TNF-α and CHX were added 1 h after T. gondii infection.

The caspase-8 inhibitor Z-IEDT-FMK (R&D Systems) (46) was dissolved in DMSO. Neutrophils were pretreated with IEDT at 100 μM or with an equivalent volume of DMSO for 30 min and infected as described above.

The cell-permeable APIM-containing peptide ATX-101 (Ac-MDRWLVKWKKKRKIRRRRRRRRRRR) (Tufts Medical School) was used to inhibit the interaction of PCNA with other proteins through the APIM motif (54). Neutrophils were treated with ATX-101 at 1, 5, or 10 μM or with an equivalent volume of DMSO as the vehicle control for 8 h.

### Immunofluorescence microscopy.

For immunofluorescence microscopy, neutrophils were settled onto coverslips coated with poly-l-lysine (Corning) for 30 min at 37°C. Infections were done for 16 h, and cells were fixed with 4% paraformaldehyde (PFA; Electron Microscopy Sciences). Neutrophils were permeabilized with 0.5% Triton X-100 (Fisher Scientific), blocked with 5% normal goat serum (Southern Biotech), and stained overnight with an anti-myeloperoxidase (MPO, N4C7[989B]; BioLegend) primary antibody, followed by an AF594-conjugated secondary antibody (Life Technologies). Coverslips were mounted onto glass slides using Vectashield with DAPI (4′,6-diamidino-2-phenylindole; Vector Laboratories). Images were acquired using a Leica SP8 confocal microscope with a 63× oil objective. Images were analyzed using FIJI (81).

### Flow cytometry.

Cells were stained with annexin V-AF647 (5 μl; Invitrogen) and 7-AAD (5 μl; BioLegend) in 100 μl of annexin-binding buffer (10 mM HEPES, 140 mM NaCl, and 2.5 mM CaCl_2_, pH 7.4) for 15 min at RT. After the incubation period, 400 μl of annexin-binding buffer was added. Infection efficiency was determined based on GFP fluorescence. Events were acquired on a FACSCalibur flow cytometer with CellQuest software (BD Bioscience) and analyzed using FlowJo software (TreeStar).

### Western blotting.

Whole-cell lysates were generated by the addition of 2× Laemmli buffer containing 10% β-mercaptoethanol to the cell pellets. Cell lysates were separated by SDS-PAGE and transferred to polyvinylidene difluoride (PVDF) membranes (Bio-Rad). Membranes were blotted with the following antibodies: anti-caspase-8 (1C12; Cell Signaling), anti-caspase-3 (8G10; Cell Signaling), anti-PCNA (D3H8P; Cell Signaling), and anti-β-actin (AC-15; Sigma-Aldrich). Peroxidase-conjugated secondary antibodies were used (BioLegend). Membranes were developed using enhanced chemiluminescence (ECL) (Thermo Scientific) and detected using a Nikon camera as previously described (82). Quantification analysis of blots was performed using ImageJ, and the β-actin signal was used as a control for normalization. Values are expressed as a percentage of the normalized signal from the mock-infected group.

### Caspase-8 activation assay.

Active caspase-8 was quantified by using a FAM-FLICA (fluorochrome-labeled inhibitor of caspase) detection kit (FAM-LETD-FMK; ImmunoChemistry Technologies) according to the manufacturer’s instructions. Events were acquired on an imaging flow cytometer (Amnis ImageStream Mark II) and analyzed using IDEAS software.

### RNA isolation and library preparation.

Four independent experiments in which neutrophils were mock infected or infected with T. gondii for 4 h were performed. Total RNA from each set of experimental samples was isolated using the Qiagen RNeasy kit (Qiagen). RNA concentration and integrity were determined using an Agilent 2100 Bioanalyzer (Agilent Technologies). One hundred nanograms of total RNA was treated with 1 U of DNase I (New England BioLabs) at 37°C for 10 min and incubated with RNAClean XP beads (Beckman Coulter). Next, poly(A) mRNA was purified using Illumina’s oligo(dT) beads, followed by fragmentation at 94°C for 8.5 min to yield a median insert length of 155 nucleotides (nt). Libraries were prepared using the TruSeq RNA library prep kit (v2) according to the manufacturer’s instructions. Each library was prepared with a unique indexed primer for multiplexing. In order to ensure proper sizing, quantitation, and quality prior to sequencing, libraries were analyzed using the Agilent high-sensitivity DNA kit. Multiplexed libraries were sequenced in single-end 86-bp reads using the NextSeq 500 platform (Illumina).

### RNA-Seq bioinformatic analysis.

Data analysis was performed with the RNA-Seq workflow module of the systemPipeR package available from Bioconductor (83). RNA-Seq reads were demultiplexed using bcl2fastq conversion software. Sequence quality was assessed using the FastQC function and trimmed using Trim Galore with an average Phred score cutoff of 30 and a minimum length of 65 bp. Read sequences were trimmed by 10 nt at the 5′ end and 2 nt at the 3′ end to avoid poor-quality bases. Trimmed sequences were aligned to the Homo sapiens reference genome (hg38) using the TopHat2/Bowtie2 suite with the corresponding annotation file from Ensembl (Homo_sapiens.GRCh38.p10.gtf). Raw expression values in the form of gene-level read counts were generated with the summarizeOverlaps function, counting only the reads overlapping exonic regions of genes and discarding reads mapping to ambiguous regions of exons from overlapping genes. Normalization and statistical analysis of differentially expressed genes (DEGs) were performed using the edgeR package, which normalizes reads by the trimmed mean of the M-value method. DEGs were defined as those with a fold change of >1.5 and a Benjamini-Hochberg-controlled false-discovery rate (FDR)-corrected *P* value of 0.05 in a comparison with mock-infected samples. Only protein-coding genes with an average of greater than 1 read per kilobase of transcript per million mapped reads (RPKM) were included in the volcano plot (84) and further analysis. Heatmaps for gene expression represent the absolute normalized expression (transcripts per million [TPM]); the range of colors is based on scaled and centered TPM values of the entire set of genes (red represents high expression, whereas blue represents low expression) (85).

### Functional enrichment.

Functional enrichment analysis was performed to identify biological pathways, including Gene Ontology (GO) terms, using Metascape (86). Significant functional enrichment terms were defined as those with an FDR-corrected *P* value of 0.05.

### Proximity ligation assay.

A proximity ligation assay (PLA) was performed with Duolink *in situ* detection reagents orange (Sigma Aldrich) according to the manufacturer’s recommendations, with minor modifications. Neutrophils were settled onto coverslips coated with poly-l-lysine (Corning) for 30 min at 37°C, and infections were done for 16 h. Cells were fixed with 4% PFA for 15 min at RT, washed with PBS, and blocked with Duolink *in situ* blocking solution for 30 min at 37°C. Cells were then stained with mouse anti-caspase-3 [3CSP03(4.1.18), 1:100; Invitrogen] and rabbit anti-PCNA (D3H8P, 1:200; Cell Signaling) primary antibodies for 4 h at RT. Cells were washed and stained with the secondary mouse PLUS and rabbit MINUS antibodies for 1 h at 37°C. Cells were washed in Duolink *in situ* wash buffer, the ligation reaction was performed at 37°C for 30 min, and it was followed by the amplification reaction at 37°C for 100 min. Cells were washed in Duolink *in situ* wash buffer and stained with Hoechst 33342 (ImmunoChemistry Technologies) for 5 min at 37°C. Coverslips were mounted onto glass slides using the Duolink *in situ* mounting medium with DAPI. Images were acquired using a Leica SP8 confocal microscope with a 63× oil objective. Images were analyzed using FIJI (81).

### Statistics.

Statistical analyses were performed using GraphPad Instat software. Analysis of variance (ANOVA) followed by a *post hoc* Tukey test was used for comparison between means. Differences were considered significant when the *P* value was <0.05.

### Data availability.

RNA-Seq data has been deposited in the NCBI Sequence Read Archive public repository: PRJNA686822.
