# Supplementary figures and images for: Toxoplasma gondii Extends the Life Span of Infected Human Neutrophils by Inducing Cytosolic PCNA and Blocking Activation of Apoptotic Caspases
Source: mBio. 2021 Jan 26;12(1):e02031-20. doi: 10.1128/mBio.02031-20 (PMC7858050; doi:10.1128/mBio.02031-20)

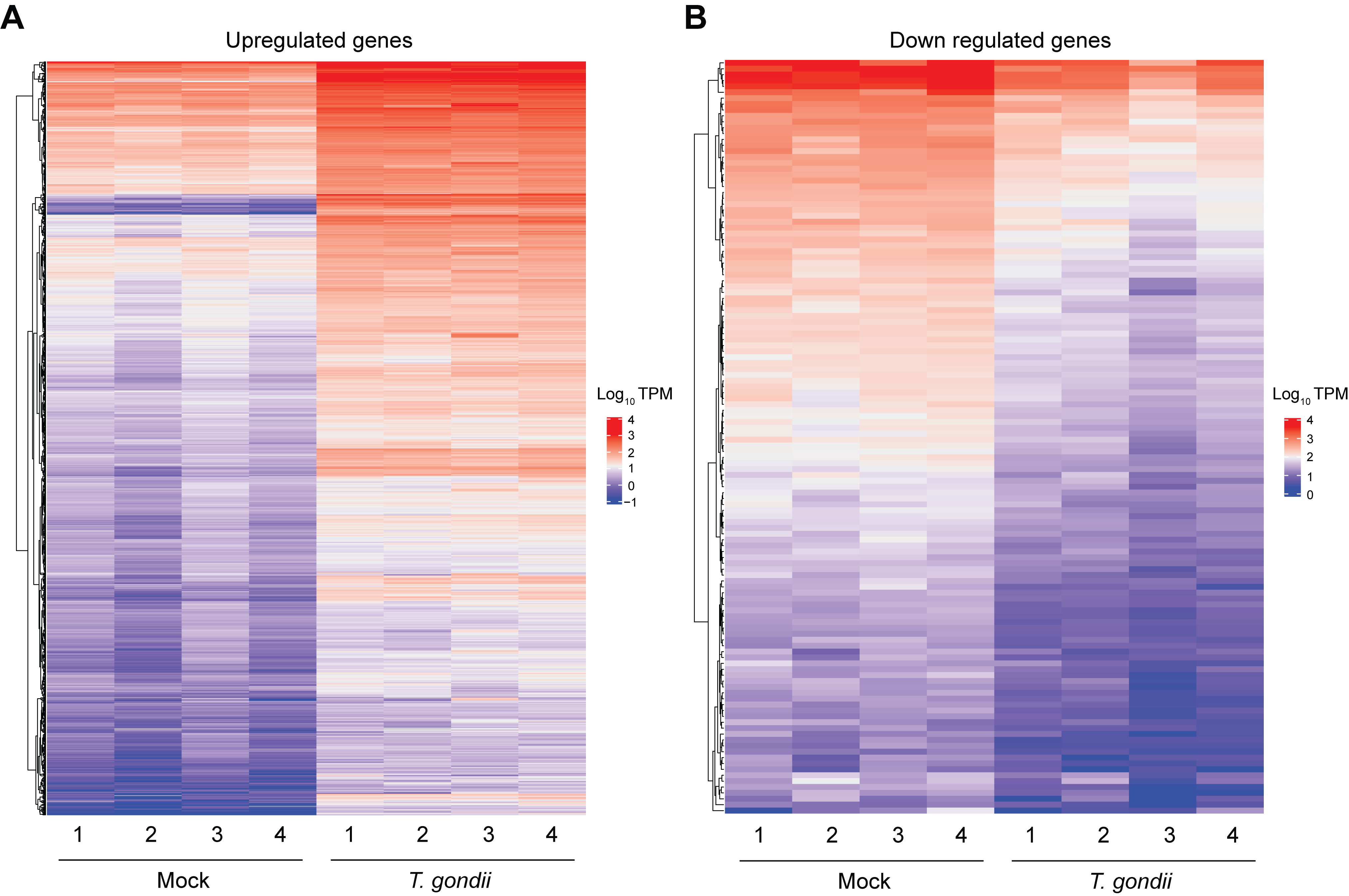

Supplement: FIG S1 [file mBio.02031-20-sf001.tif]
